# Human HER2 overexpressing mouse breast cancer cell lines derived from MMTV.f.HuHER2 mice: characterization and use in a model of metastatic breast cancer

**DOI:** 10.18632/oncotarget.19174

**Published:** 2017-07-10

**Authors:** Sunju Park, Jessie R. Nedrow, Anders Josefsson, George Sgouros

**Affiliations:** ^1^ Russell H. Morgan Department of Radiology and Radiological Science, Johns Hopkins University, School of Medicine, Baltimore, Maryland, USA

**Keywords:** HER2, radioimmunotherapy, ^111^In-DTPA-trastuzumab, breast cancer

## Abstract

Preclinical evaluation of therapeutic agents against metastatic breast cancer require cell lines and animal models that recapitulate clinical metastatic breast cancer as much as possible. We have previously used cell lines derived from the neu-N transgenic model to investigate anti-neu targeting of metastatic breast cancer using an alpha-emitter labeled antibody reactive with the rat variant of HER2/neu expressed by the neu-N model. To investigate alpha-particle emitter targeting of metastatic breast cancer using clinically relevant, commercially available anti-HER2/neu antibodies, we have developed cell lines derived from primary tumors and lung metastases from HuHER2 transgenic mice. We extracted primary mammary gland tumors, isolated the epithelial breast cancer cells, and established seven different cell lines. We also established 2 different cell lines from spontaneous lung metastases and cell lines from a serial transplantation of tumor tissues in HuHER2 transgenic mice. HuHER2 protein was overexpressed in all of the established cell lines. The mRNA level of ER (estrogen receptor) and PR (progesterone receptor) was relatively low in the cell lines compared to normal mammary gland (MG). As EMT markers, the expression of E-Cadherin in the cell lines was downregulated while the expression of TWIST1 and Vimentin were upregulated, relative to MG. Furthermore, trastuzumab directly inhibited cellular viability. Biodistribution studies with ^111^In-DTPA-trastuzumab in HuHER2 cell tumor xenografts demonstrated specific targeting with a clinically relevant antibody. Collectively, these cell lines show all the hallmarks of highly aggressive, metastatic breast cancer and are being used to evaluate combination therapy with alpha-particle emitter labeled HER2/neu reactive antibodies.

## INTRODUCTION

Current cancer treatment is largely ineffective against widely disseminated metastatic breast cancer. Stage IV breast cancer is defined by distant and widely disseminated metastases, including to the liver. Only 1 in 5 patients with stage IV breast cancer survives for more than 5 years, and patients with liver metastases have a median survival of less than 6 months [1]. Over the past several years, we have utilized a metastatic breast cancer model to investigate targeted radiopharmaceutical therapy (RPT) with alpha-particle emitters (αRPT). The neu-N model expresses the rat variant of the neu oncoprotein and syngeneic tumor cell lines derived from this model provide orthotopic models as well as metastatic models. Using syngeneically-derived cells from this model we showed that ^213^Bi-labeled anti–HER-2/neu mAb improved the survival of mice with extensive mammary carcinoma metastases [2]. In addition, we showed a metastatic progression by bioluminescent, small-animal magnetic resonance imaging, positron emission tomography, single-photon emission computed tomography (SPECT) imaging, and also by histopathology [3]. We also explored the efficiency of the murine HER2-targeted antibody modified to deliver the α-particle emitter ^225^Ac compared to the β-particle emitter ^90^Y, and found that ^225^Ac-labeled antibody significantly extended the survival in the neu-N mice with metastatic disease [4]. Finally, we recently demonstrated in this immunocompetent model that we are able to image the programmed cell death ligand 1 (PD-L1), which is part of the immune checkpoint system. However, the murine HER2-targeted antibody used for these studies and the neu-N model, is not cross-reactive with the human HER2/neu receptor making the evaluation of clinically relevant HER2-targeted antibodies such as trastuzumab and pertuzumab, impossible.

The HuHER2 transgenic mouse model expresses human HER2 under the murine mammary tumor virus (MMTV) promoter (MMTV.f.HuHER2 (Fo5) from Genentech). Finkle et al. [5] found mammary adenocarcinomas and metastatic progression frequently in female founders and progeny of HuHER2 transgenic mice at 28 weeks. Furthermore, in mice treated with mu4D5, a humanized murine version of trastuzumab, tumor growth was inhibited through inactivation of the HER2 signaling pathway.

In this work we demonstrated reactivity of HuHER2 transgenic mouse model-derived tumor cell lines with commercially available antibodies (e.g., trastuzumab (Herceptin®) and pertuzumab (Perjeta®)) that are clinically relevant and that have been used in patient therapy. We have established and characterized several syngeneic cell lines from spontaneous tumors, including lung metastases and serial transplantation of tumor tissues in HuHER2 transgenic mice. We also demonstrate that a clinically available HER2-targeted antibody, trastuzumab, can target isografts of the developed cell lines via ^111^In-DTPA-trastuzumab.

## RESULTS

### HER2 is highly expressed in the epithelia of spontaneous tumors derived from HuHER2 transgenic mice

**Figure 1:**
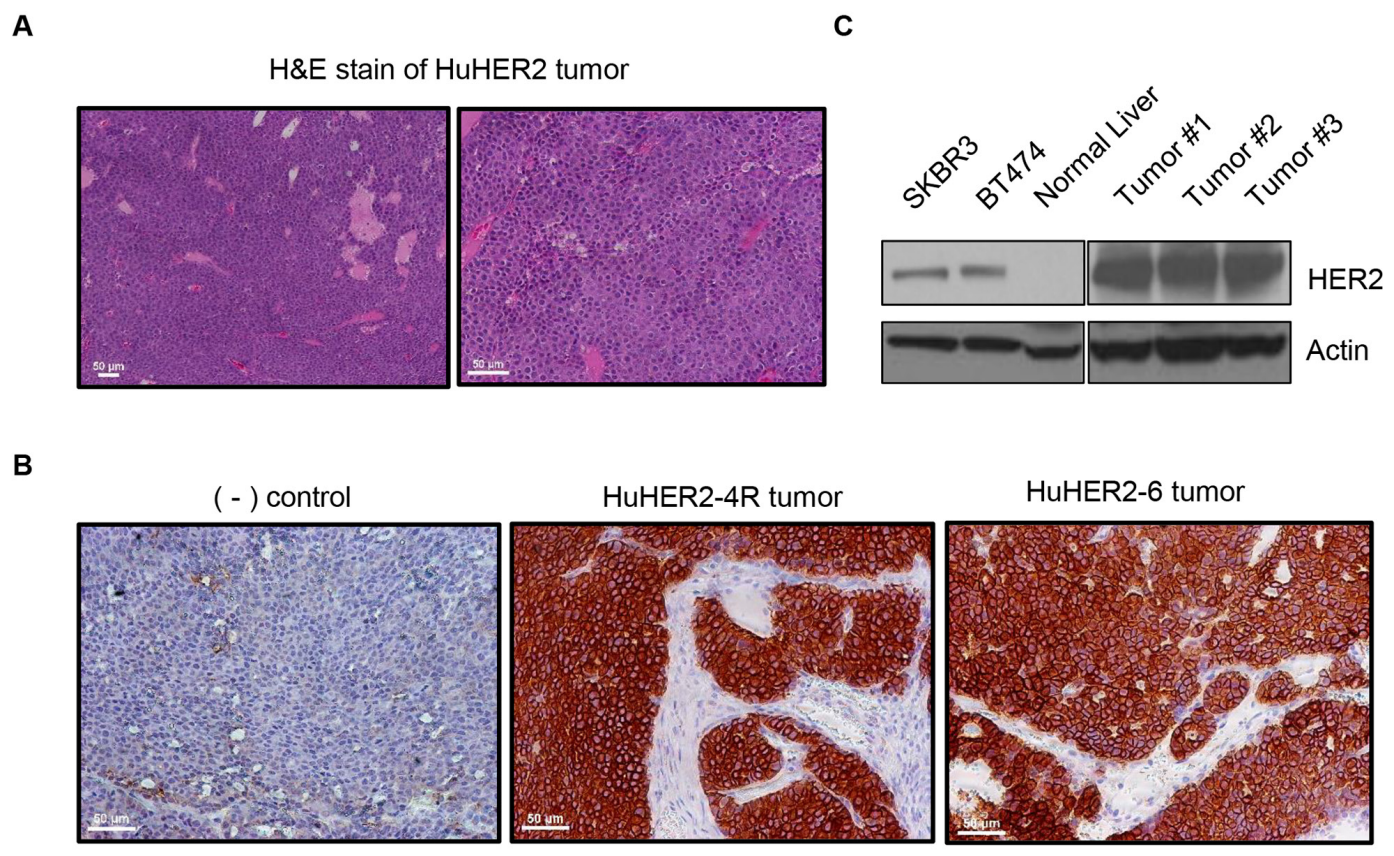
Characterization of spontaneous tumors derived from HuHER2 transgenic mice **(A)** H&E staining of a spontaneous HuHER2 tumor derived from HuHER2 transgenic mice. Shown here is a composite of 10x (left) and 20x (right) imaging. **(B)** Immunohistochemistry (IHC) and (C) Western blot analysis of HER2 in tumors. SKBR3 and BT474 were used as a HER2 positive control cell lines. Scale bars represent 50 μm.

Spontaneous mammary gland tumors developed within 6 to 12 months in HuHER2 transgenic mice (Supplementary Figure 1). Histopathological analysis with hematoxylin and eosin showed a malignant tumor consisting of layers of cells in mammary gland and invasion of HuHER2 tumor into stroma and muscle (Figure 1A), which indicated the mammary adenocarcinoma in HuHER2 transgenic mouse. Furthermore, in order to examine the HER2 expression at the cellular level in the tumor, tissue sections were prepared for immunohistochemical (IHC) analysis. Tumors were fixed, embedded in paraffin, and stained with anti-human HER2 antibodies. We found strong membranous staining of HER2 in multiple samples of tumor tissues consistent with previous reports [5] (Figure 1B). Immunoblotting analysis furthered confirmed that HER2 was strongly expressed in the tumor compared to normal tissue such as the liver (Figure 1C). Together, these results support that human HER2 is highly expressed at the surface of cells in mammary tumor tissue of HuHER2 transgenic mouse.

### Characterization of HuHER2 cell lines

**Figure 2:**
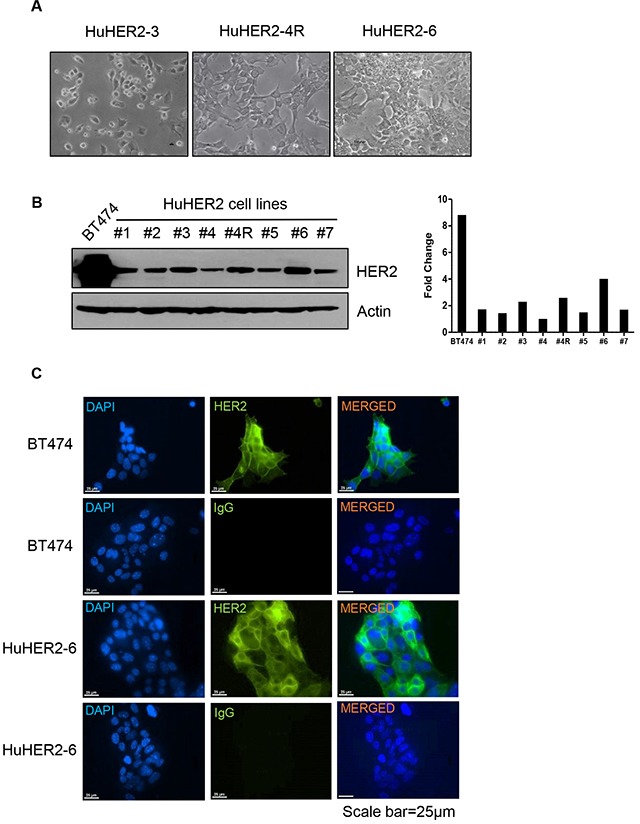
Characterization of HuHER2 cell lines overexpressing human HER2 **(A)** Morphology of representative HuHER2 cell lines. **(B)** Western blot analysis of HER2 using anti-HER2 antibody in HuHER2 cell lines. **(C)** Immunofluorescence analysis of HER2 using anti-HER2 and IgG antibody as a isotype control in HuHER2-6 and BT474 cells as a positive control.

In order to establish the HuHER2 cell line origi-nated from mammary carcinoma in HuHER2 transgenic mouse, the tumors were resected and minced for *in vitro* culture. The epithelial cells were cultured and isolated until fibroblasts were removed. As a result, we established seven different HuHER2 cell lines. Using a phase contrast microscopy, we observed that the cell lines showed epithelial-like morphology with polygonal shape in culture (Figure 2A). To investigate HER2 expression in seven different cell lines, we performed the immunoblotting using anti-HER2 antibody. We found that HER2 protein was highly expressed in all of the isolated cell lines. One of the cell lines (denoted HuHER2-6) had 4 times more protein expression compared to the other 6 cell lines developed. (Figure 2B and Supplementary Figure 2A). To further examine a localization of HER2 expression in HuHER2-6 cell line, we performed an immunofluorescence staining analysis using anti-HER2 antibody and FITC conjugated trastuzumab, a monoclonal antibody that interferes with the HER2 receptor for the clinical application. We found that HER2 membranous staining was similar to that of BT474, a high HER2 expressing cell line as a positive control. (Figure 2C and Supplementary Figure 2B).

**Figure 3:**
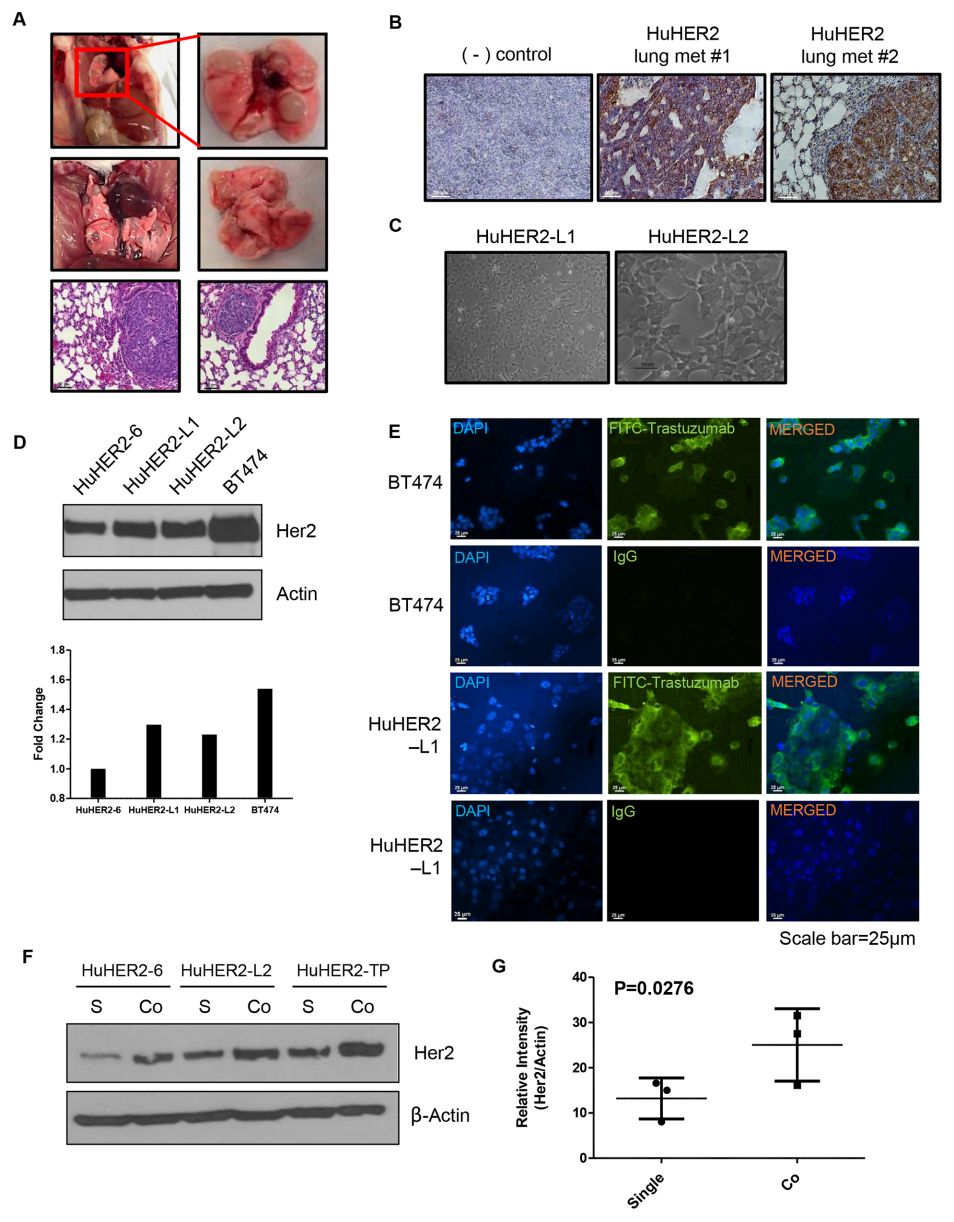
Characterization of spontaneous lung metastases in HuHER2 transgenic mice and establishment of HuHER2-Lung metastasis cell lines overexpressing human HER2 **(A)** Representative gross photo of spontaneous lung metastases and corresponding histological (H&E stained) images. **(B)** IHC analysis of HER2 in lung metastases. **(C)** Morphology of established HuHER2 lung metastasis cell lines (HuHER2-L1 and L2). **(D)** Western blot analysis of HER2 using anti-HER2 antibody in HuHER2-L1 and L2 cell lines. **(E)** Immunofluorescence analysis of HER2 in HuHER2-L1 cell line by FITC conjugated trastuzumab. **(F)** Western blot analysis of HER2 in single or co-culture of HuHER2 cell lines with cancer associated fibroblast derived from HuHER2 tumors. **(G)** Mean density value of HER2 compared to that of total actin.

We observed spontaneous lung metastases with high HER2 expression at approximately 40 weeks (Figure 3A and 3B). Using these lung metastases, we also established two different cell lines such as HuHER2-L1 and-L2. The morphology of these cell lines is similar to those derived from the spontaneous mammary tumors (Figure 3C). HuHER2-L1 and L2 exhibited approximately 1.3-fold greater HER2 protein expression than HuHER2-6 (Figure 3D). To further investigate HER2 expression in cell membrane, we utilized FITC-conjugated trastuzumab for the immunofluorescence staining and found that strong membranous staining for HER2 in HuHER2-L1 cell line as HuHER2-6 cell line (Figure 3E). Therefore, these finding suggest that established HuHER2 cell lines have a significant HER2 expression on the cell surface and are, therefore, targetable by anti-HER2 antibodies such as trastuzumab.

### Co-culture of HuHER2 cells with fibroblasts promotes HER2 expression

Crosstalk between tumor cells and tumor micro-environment in many tumor types can increase tumor growth and metastasis [6, 7]. We found that HER2 expression was higher in tumor tissue than in the HuHER2 cell lines. In order to investigate if HER2 expression can be increased by stroma, we co-cultured HuHER2 cells with isolated cancer associated fibroblasts (CAFs) from the spontaneous tumors. Co-culturing the different cell types enhanced HER2 expression about 2-fold compared to the single cultures (Figure 3F and 3G). These results suggest that the crosstalk between HuHER2 tumor cells and CAFs increases HER2 expression in mammary tumor tissue.

### Phenotypic markers of HuHER2 cell lines

**Figure 4:**
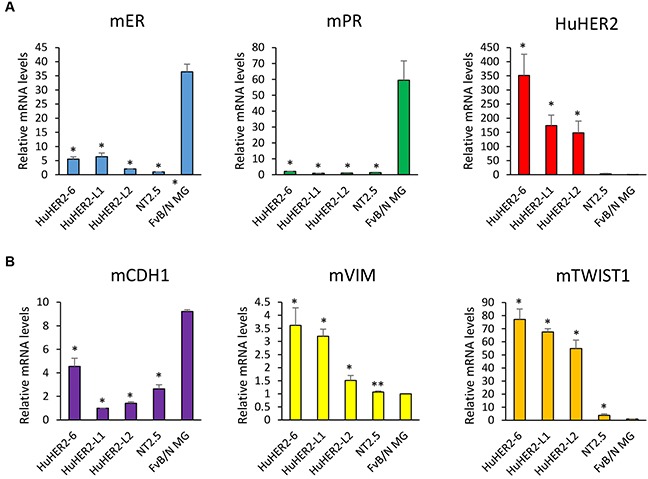
Expression of mouse ER, mouse PR, human HER2 and mouse EMT markers in HuHER2 cell lines **(A)** Real time qRT-PCR was performed to measure mRNA levels of mouse ER, PR, human HER2 and **(B)** mouse EMT markers (CDH1, Vimentin and TWIST1) in HuHER2 cell lines. Normal mammary gland tissue and NT2.5 mouse cells expressing rat HER2 were used as controls. (* P< 0.005, **P<0.05).

Breast cancer aggressiveness is typically char- acterized by evaluating expression of estrogen and progesterone hormone receptors (ER and PR, respectively). Low expression of ER and PR indicates a breast cancer phenotype that is less susceptible to conventional therapeutics [8]. Given the robust HER2 expression in HuHER2 cell lines, we identified a breast cancer subtype of HuHER2 cell line by measuring the expression of ER, PR using real time quantitative RT-PCR. We found that HuHER2-6 and HuHER2-L2 ER mRNA levels were 15% and 5% of the level measured in normal mammary gland (MG), respectively. PR mRNA was also substantially lower (< 3%) relative to normal MG while the human HER2 mRNA was higher in HuHER2-6, HuHER2-L1 and L2 (Figure 4A).

The propensity of breast cancer for metastatic dissemination is positively related to expression of Epithelial–Mesenchymal Transition (EMT) markers such as Twist1 and Vimentin, and negatively related to expression of adhesion markers such as E-Cadherin. These were evaluated by real time quantitative polymerase chain reaction (QRT-PCR). We observed that the expression of E-Cadherin (CDH1) that is known as downregulation in EMT was decreased about 15% in HuHER2-L1 and –L2 compared to MG tissue. The expression of both Vimentin and TWIST1 that are known as upregulation in EMT were elevated 3-fold and 70-fold, respectively, relative to MG (Figure 4B). Collectively, the subtype of HuHER2 cell line might be a human HER2 positive mouse breast cancer with ER and PR negative. In addition, HuHER2 cell line show all the hallmarks of highly aggressive, metastatic breast cancer.

### HER2 dependent HuHER2 cell growth

**Figure 5:**
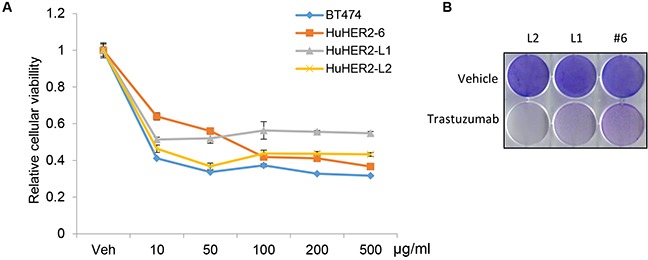
Cellular viability assay of HuHER2 cell lines exposed to trastuzumab **(A)** MTT assay was performed in BT474, HuHER2-6, HuHER2-L1 and L2 cells treated with 10 to 500 μg/ml of trastuzumab for 3 days. **(B)** Crystal violet staining assay of HuHER2-6 (#6), HuHER2-L1 (L1) and HuHER2-L2 (L2) cells treated with 50 μg/ml of trastuzumab for 3 days. Cells were stained with crystal violet solution for 10 min and representative results are shown.

To assess the sensitivity to trastuzumab in the growth of HuHER2 cell lines, *in vitro*, we performed cellular viability analysis using HuHER2 cell lines treated with concentrations of trastuzumab ranging from 10 to 500 μg/ml. The cellular viability was decreased to 50% at 10 to 50 μg/ml of trastuzumab (Figure 5A and 5B). Furthermore, we observed that 5, 7, and 10 ug/ml of trastuzumab sensitized HuHER2-L1 and L2 cell lines compared to HuHER2-6 and the high HER2 expressing BT474 cells was more sensitive to trastuzumab than HuHER2 cell lines. Thus, we demonstrated that the inhibitory effect of trastuzumab on cellular viability of HuHER2 cell lines is significantly correlated with the amount of HER2 expression (Supplementary Figure 3). The result suggested that inhibition of the HER2 dependent pathway is apparently critical for the survival of HuHER2 cell line. Thus, HuHER2 cell line might be a beneficial to test a functional HER2 antagonism.

### Biodistribution of ^111^In-DTPA-trastuzumab in HuHER2 cell tumor bearing HuHER2 transgenic mice

To enable *in vivo* evaluation of therapeutic response to anti-HER2 therapy, we established a luciferase reporter stable HuHER2-L2 (HuHER2-L2-Luc+) cells and injected the cell subcutaneously into athymic nude mice. The result showed that HuHER2-L2-Luc+ cell tumor growth was detected within 10 days after injection using the IVIS imaging (Supplementary Figure 4).

Next, we evaluated the feasibility of targeting tumors derived from HuHER2-L2-Luc+ cells by evaluating the biodistribution of intravenously administered trastuzumab, labeled with ^111^In [9, 10]. The distribution of ^111^In-DTPA-trasuzumab was achieved by *ex vivo* counting of tissues collected from mice sacrificed at 24 hours after the p.i. (post injection). By 24 hours, the accumulation of ^111^In-DTPA-trastuzumab peaked in the tumor at 27.19 ± 15.047%ID/g with moderate tumor to muscle/blood ratios (1.35 ± 0.551, 13.483 ± 12.182). The imaging probe’s concentration in blood, heart, lung, liver, spleen, kidney, stomach, intestine, bone, muscle and BAT (Brown adipose tissue) was 13.483 ± 12.182, 6.140 ± 2.738, 6.285 ± 3.434, 18.823 ± 5.146, 12.167 ± 7.110, 6.818 ± 2.319, 3.743 ± 2.044, 2.993 ± 1.161, 4.963 ± 2.531, 1.335 ± 0.551 and 4.550 ± 1.682%ID/g, respectively (Supplementary Figure 5).

**Figure 6:**
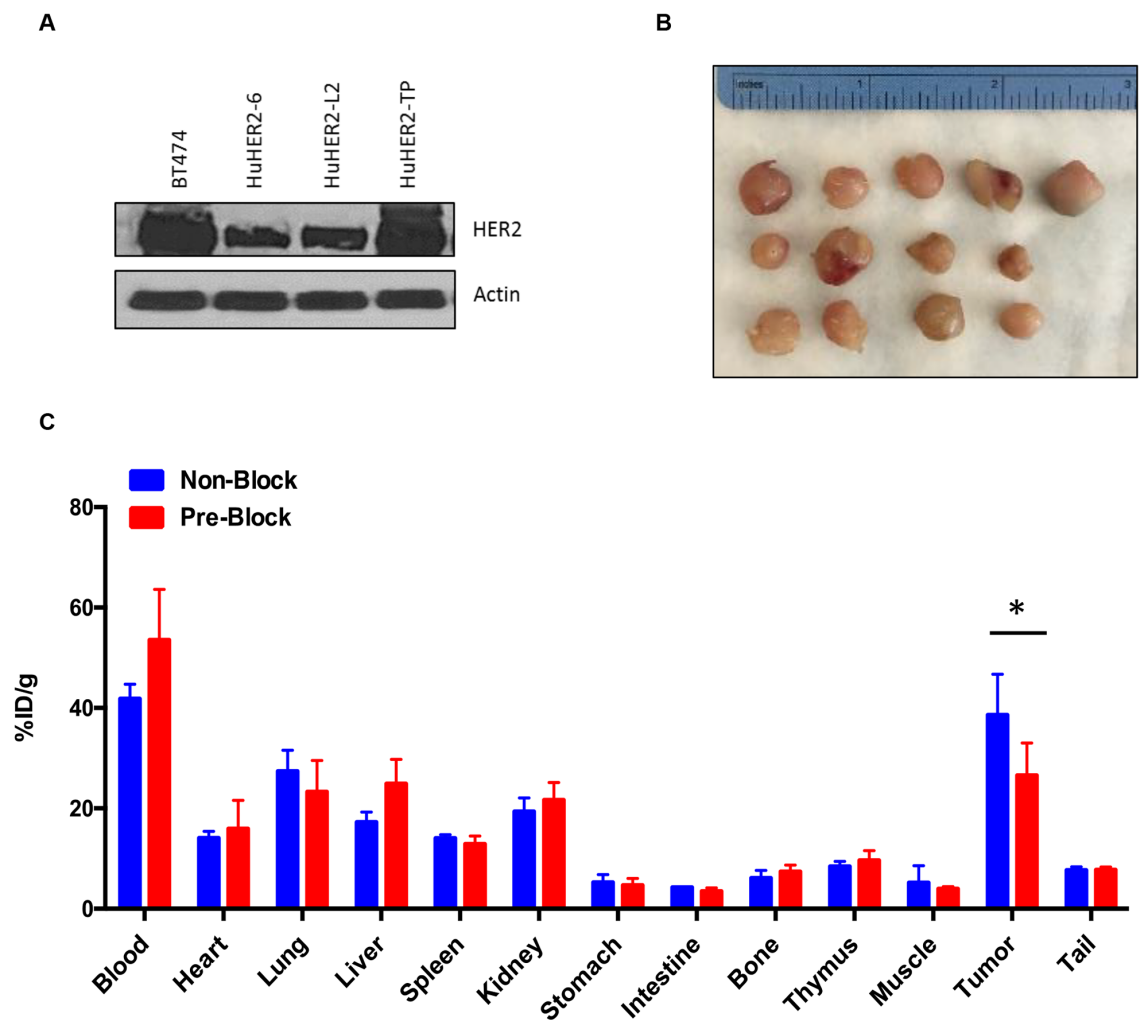
Biodistribution of 111In-DTPA-trastuzumab in HuHER2-TP cell tumor bearing HuHER2 transgenic mouse model **(A)** Western blot analysis of HER2 in HuHER2-6, HuHER2-L2 and HuHER2-TP cell lines. **(B)** Representative HuHER2-TP cell tumors in a HuHER2 transgenic mouse. **(C)** Biodistribution of ^111^In-DTPA-trastuzumab in HuHER2-TP cell-derived tumor bearing mice at 24 hours p.i. for normal tissues and tumors. Blue bar represents mice injected with ^111^In-DTPA trastuzumab only. Red bar represents mice pre-treated with cold trastuzumab 24 hours prior to ^111^In-DTPA trastuzumab injection. (* P< 0.04).

Serially transplanted tumor help enhance the propensity for metastatic dissemination [11]. To create the HuHER2 cell line that have a metastatic phenotype and can lead spontaneous metastasis ultimately to various distant organs, we established a cell line (HuHER2-TP) by serially transplanting a spontaneously developed mammary tumor into the mammary fat pad and interestingly, found that HER2 expression was upregulated in HuHER2-TP cells compared to HuHER2-6 and HuHER2-L2 (Figure 6A, 6B and Supplementary Figure 6). Furthermore, to examine a competitive effect of ^111^In-DTPA-trastuzumab, we developed tumors of HuHER2-TP cells in HuHER2 transgenic mice (Figure 6B) and performed the biodistribution analysis with ^111^In-DTPA-trastuzumab. At 24 hours post-injection of ^111^In-DTPA-trastuzumab the HER2 positive tumor demonstrated high uptake (38.6 ± 8.1 %ID/g). The radiolabeled antibody still had significant uptake in the blood (41.8 ± 2.9 %ID/g) as evident by the tumor to blood ratio of 1.0 ± 0.2, which is typical of radiolabeled antibodies at early time points. However, the higher tumor to muscle ratio 8.6 ± 2.6 easily distinguished the tumor from the background. The remaining tissue %ID/g are as follows: heart (14.1 ± 1.3), lung (27.4 ± 4.1), liver (17.2 ± 2.0), spleen (14.1 ± 0.7), kidney (19.4 ± 2.7), stomach (5.2 ± 1.6), intestine (4.3 ± 0.12), bone (6.1 ± 1.6), thymus (8.4 ± 0.9), and tail (7.7 ± 0.60), respectively (Figure 6C). Tumor binding specificity was demonstrated by showing a significant decrease (p ≤ 0.04) in tumor uptake (26.6 ± 6.5 %ID/g) when excess trastuzumab was administered 24 hours prior to ^111^In-DTPA-trastuzumab (Figure 6C). These results confirm that the clinically relevant anti-HER2 antibody, trastuzumab, is able to localize to an orthotropic mouse model using the developed HuHER2 cell lines by targeting their HER2 expression.

## DISCUSSION

The transgenic mouse models differ from conventionally used xenograft model systems in a number of important and fundamental ways. Breast cancer transgenic models develop breast cancer spontaneously with a higher frequency than normal mice. Since the tumors are naturally occurring, they are more similar to the development and progression of human breast cancer tumors. Furthermore, the transgenic models are immunocompetent, making it possible to evaluate potential immune responses to conventional therapies as well as αRPT. Furthermore, these immunocompetent models can be utilized to evaluate novel therapies focused on the immune checkpoints [12, 13]. However, although transgenic mice develop breast cancer spontaneously the length of tumor development as well as the unpredictability of this process makes it impractical to rely on for therapeutic efficacy studies. To help address this issue, syngeneic tumor cell lines are typically derived for use in more conventional models such as orthotropic tumor models. Orthotropic injection of a syngeneic tumor cell lines derived from the spontaneous tumors of transgenic mice, provide more consistent and rapid tumor growth at a site that matches the original microenvironment niche [14, 15].

Our previous works investigated αRPT using an analogous transgenic model (neu-N) with a corresponding syngeneic tumor cell line (NT2.5) demonstrated increased survival in mice with metastatic breast cancer. These studies showed that αRPT has the potential to be highly effective against metastatic disease. This metastatic breast cancer model is based on the dissemination of NT2.5 cells via a left cardiac ventricle (LCV). However, NT2.5 cells are derived from spontaneous tumors in the neu-N mouse model that express the rat version of HER2*/*neu [2–4]. Thus, requiring a murine antibody in these investigations that is not cross-reactive with the human HER2/neu receptor. Nonetheless, our works demonstrated the feasibility of αRPT against disseminated metastases and also identified cross-reactive normal organs. However, the further investigation of a human HER2-targeted αRPT agent in an immunocompetent mouse model has the potential to help translate this therapy to the clinic.

In this study, we used a HuHER2 transgenic model that is more closely related to human breast cancer [5]. The HuHER2 model is an immunocompetent transgenic mouse model that expresses the human variant of the neu oncoprotein. Currently, this model has been used to investigate resistance to trastuzumab therapy. Using a mouse model of HER2-overexpressing (HER2+), PIK3CA (H1047R)-mutant breast cancer, PIK3CA (H1047R) enhances HER2-mediated breast epithelial transformation and metastatic progression, which develops trastuzumab resistance [16]. From this model, we have established and characterized syngenic cell lines derived from the model’s spontaneous breast cancer tumors. Furthermore, we have evaluated the potential of these derived syngeneic cell lines in an orthotropic model to investigate clinically relevant HER2-targeted antibody conjugates.

We established seven different HuHER2-derived syngeneic cell lines from spontaneous mammary tumors as well as two lung metastatic cell lines all of which highly expressed human HER2. The low expression of ER and PR in these HuHER2-derived cell lines places them in the HER2 positive, ER and PR negative breast cancer subtype. Such cancers are less likely to respond to hormone modulation therapy. We also established a cell line with a more aggressive phenotype (HuHER2-TP) through the serial transplantation of tumor tissue. It is well known that serially transplanted tumors progress from a noninvasive, low-grade cancer to a higher-grade invasive disease [11]. Thus, the HuHER2-TP cell line might be a useful model to study for metastatic breast cancer for the future study.

Through the QRT-PCR, we found that upregulation of Vimentin and Twist1 as EMT (epithelial-mesenchymal transition) markers, and down regulation of the adhesion marker E-cadherin [17] suggests that the HuHER2 cell lines have a more aggressive, metastatic phenotype.

We confirmed that the clinically relevant anti-HER2 antibody, trastuzumab, was active against the HuHER2-derived cells. We observed significantly decreased viability of these cell lines following incubation with clinically relevant concentrations of trastuzumab. We also showed selective uptake of ^111^In-labeled trastuzumab, ^111^In-DTPA-trastuzumab, in an orthotropic model utilizing our HuHER2-derived cells. These agents have been utilized in clinical studies. Gaykema et al. investigated the effect of trastuzumab treatment on ^111^In-trastuzumab uptake and found trastuzumab treatment decreases tumor ^111^In-trastuzumab uptake around 20% [18]. Furthermore, Perik et al. found that ^111^In –labeled trastuzumab scintigraphy is not helpful to predict trastuzumab-related cardiotoxicity in metastatic breast cancer patients, but it is valuable to identify HER2-positive tumors [19]. The specific *in vivo* binding of trastuzumab in mice with tumors derived from the HuHER2 cell lines as well as the tumor to muscle- and tumor-to-blood ratios are consistent with the level of tumor targeting seen in human tumor xenograft models. These results utilizing clinical agents are supportive of the feasibility of these cells to be utilized in an immunocompetent mouse model expressing human HER2.

In contrast to xenograft models, transgenic models provide a background of cross-reactive tissue, similar to that observed in most human tumor-associated antigen targeting. Although there are advantages to using human tumor xenografts to investigate therapeutic responses to drugs, important aspects of the immune response are difficult to examine because of the absence of critical immune surveillance elements – T cells in athymic mice and both B- and T-cells in severe combined immunodeficient (SCID) mice. Humanization of these immunodeficient models has been explored. NOD/SCID mice can be transplanted with peripheral blood or bone marrow cells, inducing an anti-tumor immune response. However, there are two major disadvantages to overcome. First, it is extremely difficult to restore an immune system completely due to HLA (human leukocyte antigen) class restriction. In addition, it is quite long process that the irradiated new born mice are injected with human hematopoietic stem cells from human umbilical cord blood [20]. It is far simpler and a better mimic to the development and progress of cancer in humans, to have animal models with intact immune systems.

To summarize, we have developed and characterized HER2-expressing cell lines derived from a HuHER2 transgenic mouse model. The cells are ER and PR-negative and exhibit phenotypic characteristics that are consistent with aggressive breast cancer that has a high propensity for metastatic dissemination. We expect that this model will be used for therapeutic radioimmunoconjugate studies targeting a clinically relevant antigen in an immunocompetent mouse model.

## MATERIALS AND METHODS

### Reagents

All chemicals were purchased from Sigma-Aldrich Chemical Co. (St. Louis, MO, USA) or Thermo Fisher Scientific (Pittsburgh, PA, USA), unless otherwise specified. Aqueous solutions were prepared using ultrapure water (resistivity, 18 MΩ.cm) treated with Chelex resin purchased from Bio-Rad Laboratories, Inc. (Berkeley, CA, USA). p-SCN-Bn-DTPA was purchased from Macrocyclics, Inc. (Dallas, TX, USA). Indium-111 ([^111^In] InCl_3_) was purchased from MDS Nordion (Vancouver, BC, Canada). Trastuzumab (Herceptin), an anti-human HER-2 IgG monoclonal antibody was obtained from Genentech (San Francisco, CA, USA).

### Establishment of HuHER2 cell lines

Using aseptic techniques a small incision in the outer skin was made to expose subcutaneously growing tumor near the mammary fat pad. The tumor was carefully extracted using scissors while minimally affecting adjacent tissue. After assuring that there is no persistent bleeding, the skin incision was closed by metal clips under isoflurane and oxygen inhalation anesthesia. Wound clip was removed at 1-2 weeks. Tumor tissue was put on a petri dish over ice and a small amount of media was added and tissue was minced by razor blade. Media was added as necessary to make sure the tissue did not dry out. The pieces were transferred to a 15 ml tube. Media containing digestive enzymes (collagenase and hyaluronidase) was added to the minced tissue. The tissue was digested by rotating in a Hybaid oven at 37°C for at least one hour. When digestion was completed the digestive enzymes were inactivated by additional media. The mixture was centrifuged at 1500 rpm for 5 minutes at 4°C. Red blood cells were removed by serial resuspension and centrifugation using lysis buffer. Fibroblast cells were removed by brief trypsin incubation during several passages.

### Cell lines and constructs

SKBR3 and BT474 cell lines were obtained from the American Type Culture Collection (Manassas, VA, USA). pGL4.50[luc2/CMV/Hygro] vector was purchased from Promega (Madison, WI, USA) and transfected into the cell line using Lipofectamin 2000 as describe in the manufacture’s protocol. Luciferase reporter stable cell line was selected by 25 μg/ml of Hygromycin B (Thermo fisher).

### Identification of HuHER2 transgenic mice

Mice (2-6 months) were bred as both a monogamous pair (one adult male and one adult female) and a polygamous trio (one adult male and two adult females) breeding scheme. Offspring were weaned at 21 days. On the day of weaning, a small piece of tail (5mm or less) was snipped with sterile scissors and the DNA was extracted using STE lysis buffer. 0.5 μl of DNA was used for genotyping via PCR. A primer set specific to human growth hormone exons 4 and 5 (forward -5’-CCTCCTAAAGGACCTAGAGGAAGGC-3’ and reverse -5’-CAAGGCCAGGAGAGGCACTGGGGAG-3’) was used [5].

### Ex vivo biodistribution studies

Biodistribution studies were performed using athymic nude mice bearing HuHER2-L2-Luc+ tumors and HuHER2 transgenic mice [5] bearing HuHER2-TP tumors. Biodistribution experiments were conducted as previously described with minor modifications [2, 10, 21]. Briefly, healthy female mice (8-to-12-weeks) were sub-cutaneously (s.c.) injected in the mammary fat pad with 1 × 10^6^ cells in matrigel. 3 weeks post-tumor implantation, mice (n=4) were injected intravenously with ^111^In-DTPA-trastuzumab (0.37 MBq, 0.5 antibody protein [mg]/mouse mass [kg]). In addition, HuHER2 transgenic mice (n=4) were pre-blocked with excess unlabeled antibody 24 hours prior to the administration of ^111^In-DTPA-trastuzumab. At 24 hours post injection (p.i) of ^111^In-DTPA-trastuzumab the mice were sacrificed. The blood, heart, lungs, liver, kidneys, spleen, stomach (with content), intestine (with content), bone, thymus, muscle, tail, and tumors were harvested, weighed, and measured for radioactivity in a gamma well counter (Perkin-Elmer 2470 WIZARD^2^® Automatic Gamma Counter, Waltham, MA, USA). The percent-injected dose per gram (%ID/g) was calculated by comparison to a weighed, diluted standard. All animal studies were approved by the Animal Care and Use Committee of the Johns Hopkins University, School of Medicine.

### Real-time qRT-PCR

Total RNA was extracted with Trizol reagent (Invitrogen), and cDNA was synthesized from total RNA using an iScript Reverse Transcriptase (Biorad). Aliquots of cDNA were used as templates for real-time RT-qPCR procedure using specific primer (mouse ER Forward: 5’ TTTCTGTCCAGCACCTTGAA 3’, Reverse: 5’ CCAGGAGCAGGTCATAGAGG 3’, mouse PR Forward:5’ GGTGGAGGTCGTACAAGCAT 3’, Reverse: 5’ CTCATGGGTCACCTGGAGTT 3’, humanHer2 Forward: 5’ CCTGCCCTCTGAGACTGATG 3’, Reverse: 5’CAAGTACTCGGGGTTCTCCA 3’, mouse Ki67 Forward: 5’ TGTGGAAGAGCTGGTTAGCA 3’, Reverse: 5’ CCTGGGAGGCAGTCTTCATA 3’, mouse CDH1 Forward: 5’ CCTGCCAATCCTGATGAAAT 3’, Reverse: 5’ GTCCTGATCCGACTCAGAGG 3’, mouse TWIST1 Forward: 5’ CGGACAAGCTGAGCAAGATT 3’, Reverse: 5’ ATCCTCCAGACGGAGAAGG 3’, mouse VIM Forward: 5’ AAGGAAGAGATGGCTCGTCA 3’, Reverse:5’ TTGAGTGGGTGTCAACCAGA 3’). Relative quantities of mRNA expression were analyzed using real-time qPCR (Applied Biosystem 7500 Real-Time PCR system, Applied Biosystems, Grand Island, New York, USA). The Maxima SYBR Green/ROX Master Mix (Fermentas, Pittsburgh, Pennsylvania, USA) was used according to the manufacturer’s instruction.

### Western blotting

Cells were seeded in 60 mm dishes and had grown to 80% confluence. Cells were rinsed with a cold PBS (3x) and lysed with RIPA buffer with protease inhibitor on ice for 30 minutes. The lysates were centrifuged at 13,000 rpm for 15 minutes. The supernatant was collected and quantified by BCA assay. The same amount of extracted lysate was vertically electrophoresed on 4-12% Bis-Tris NuPage Novex Gel in MOPS SDS running buffer (Invitrogen), then transferred to nitrocellulose membranes. Membranes were stained with Ponceau S stain to confirm protein transfer, then blocked with 5% powdered milk in PBS with 0.2% Tween-20 (PBST) for one hour at room temperature. Membranes were probed with primary antibody (anti-Her2, cell signaling) in 3% milk/PBST overnight, rinsed three times with 1X PBST for 5 minutes, then probed with secondary antibody at 1:2000 dilution in 3% milk/PBST for 1 hour. Membranes were rinsed three times with 1X PBST for 5 minutes, and then treated with ECL Detection Reagent (GE Healthcare) for 1 minute. Membranes were exposed to Hyblot CL autoradiography film to determine protein expression. The quantification of protein level was normalized by the actin.

### Immunohistochemistry

Immunohistochemistry (IHC) staining was performed on paraffin sections using anti-Her2 antibody (Ab). Endogenous peroxide activity was quenched by 10 minutes incubation in 3% H_2_O_2_ and non-specific binding was blocked with serum. Sections were incubated with anti-Her2 Ab (cell signaling) at a 1:400 dilutions overnight. Diluted biotinylated anti-rabbit IgG (Vectastain kit) was added to the sections and incubated for 30 minutes. Vectastain ABC reagent (Vector) and 3, 30-diaminobenzamidine (DAB) was then used for staining color development. Counterstaining was performed with hematoxylin solution.

### Immunofluorescence staining

Cells on 6 well plates were fixed in 4% paraformaldehyde and permeabilized in 0.1% Triton X-100. After 1 hour block in 4% BSA + 0.3% Triton X-100 in PBS, the cells were incubated at 4°C for overnight with primary, anti-Her2 rabbit Ab and IgG control Ab (cell signaling) at 1:250 dilution. Cells were then incubated with secondary antibody conjugated to Alexa fluor 488 (Molecular Probes) for 1 hour at room temperature. The cell nuclei were stained using DAPI. The fluorescence was then observed under a microscope (Nikon 80i).

### Cellular viability assay

Cells were plated in 96-well plates at 2,500 cells per well in triplicate. New serum-free medium with 10 to 500 μg/ml concentration of Herceptin was added for 3 days. MTT solution (5 mg/mL in PBS, Amresco) was added to each well and cells incubated for 2 hours. Media was then aspirated and cells resuspended in DMSO. Absorbance at 560 nm was measured, with background at 670 nm subtracted. Triplicates were averaged for a mean absorbance. Percentage surviving was calculated as the absorbance of treated cells relative to time-matched vehicle-treated cells.

### Co-culture of HuHER2 cells with cancer associated fibroblasts (CAFs)

The HuHER2 cells and CAFs were separately seeded into 6-well (5×10^5^ HuHER2 cells/well; 2×10^5^ CAFs/insert) Transwell plates (0.4 μm pore-size; Corning Costar, St Louis, MO, USA). The co-culture system was maintained for 3 days and the HuHER2 cells were lysed with RIPA buffer and western blotting analysis was performed.

### Radiolabeling of DTPA-trastuzumab antibodies with ^111^In

The radiolabeled anti-HER2 antibody was prepared as previously described [10]. Briefly, the antibody was conjugated to *N*-[2-amino-3-(*p*-isothiocyanatophenyl) propyl]-*trans*-cyclohexane-1,2-diamine-*N,N*′,*N*′,*N*″,*N*″-pentaacetic acid (p-SCN-Bn-DTPA) and purified by size exclusion. The resulting antibody conjugate was added to an acid washed 1.5 mL Eppendorf tube containing [^111^In]InCl_3_ (37-74 MBq), 0.25 mL of 0.2 M HCl and 0.03 mL of 3 M NH_4_OAc, pH=7. The resulting mixture was allowed to set at room temperature for 45-60 minutes then purified as described previously. Radiochemical purity was determined by thin layer chromatography (TLC), and the protein concentration was determined by Nanodrop (Wilmington, DE, USA).

## SUPPLEMENTARY MATERIALS FIGURES


