# Supplementary material for: Human HER2 overexpressing mouse breast cancer cell lines derived from MMTV.f.HuHER2 mice: characterization and use in a model of metastatic breast cancer
Source: Oncotarget. 2017 Jul 10;8(40):68071–82. doi: 10.18632/oncotarget.19174 (PMC5620237; doi:10.18632/oncotarget.19174)
Supplement: Supplementary file 1 [file oncotarget-08-68071-s001.pdf]

Human HER2 overexpressing mouse breast cancer cell lines derived from MMTV.f.HuHER2 mice: characterization and use in a model of metastatic breast cancer

SUPPLEMENTARY MATERIALS

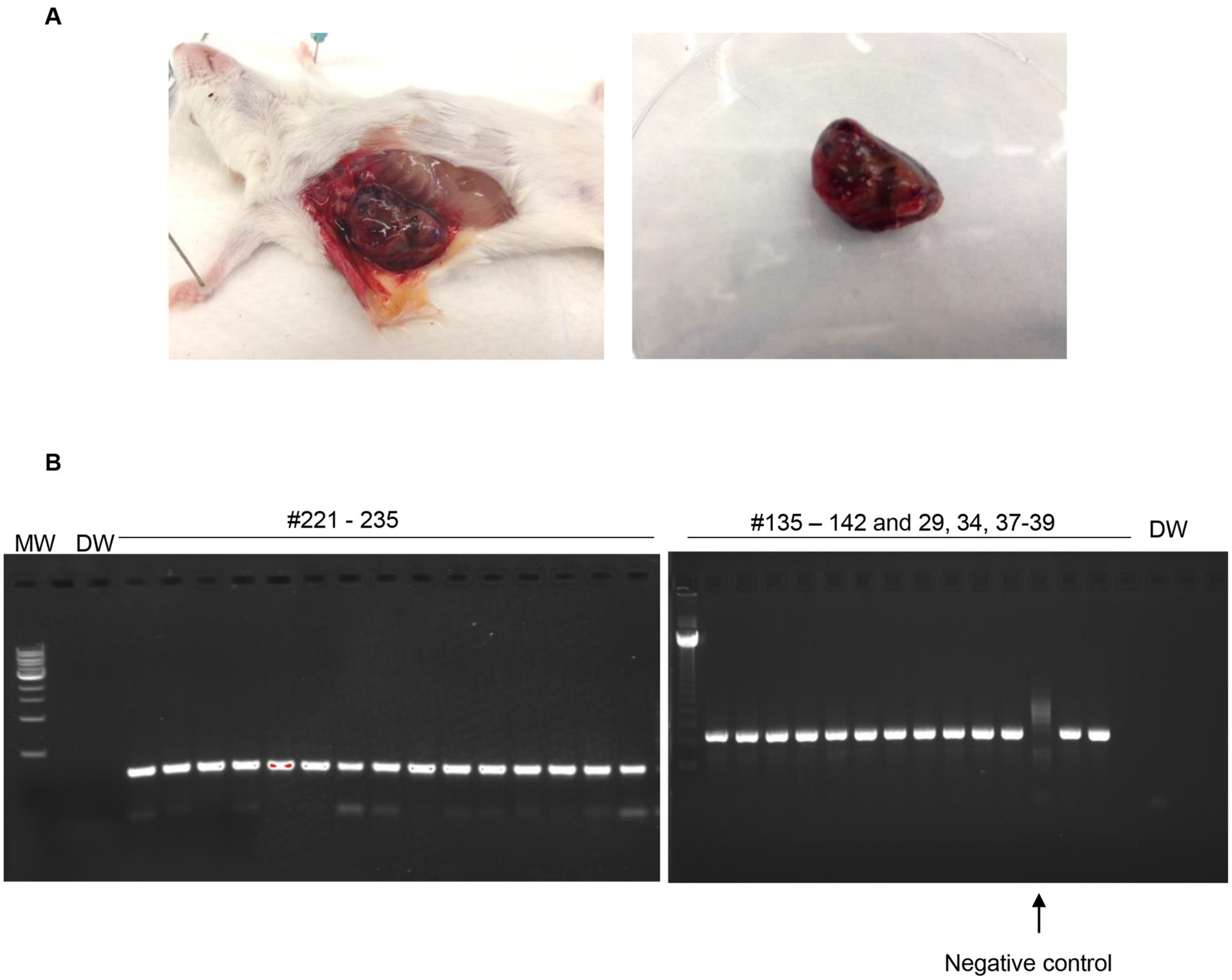

Supplementary Figure 1: Spontaneous mammary gland tumor and genotyping of HuHER2 transgenic mouse. (A) Representative photo of a spontaneous mammary gland tumor in HuHER2 transgenic mouse. **(B)** Genomic DNA was isolated from mouse tail biopsies and amplified by PCR. MW indicates molecular weight marker; DW indicates distilled water.

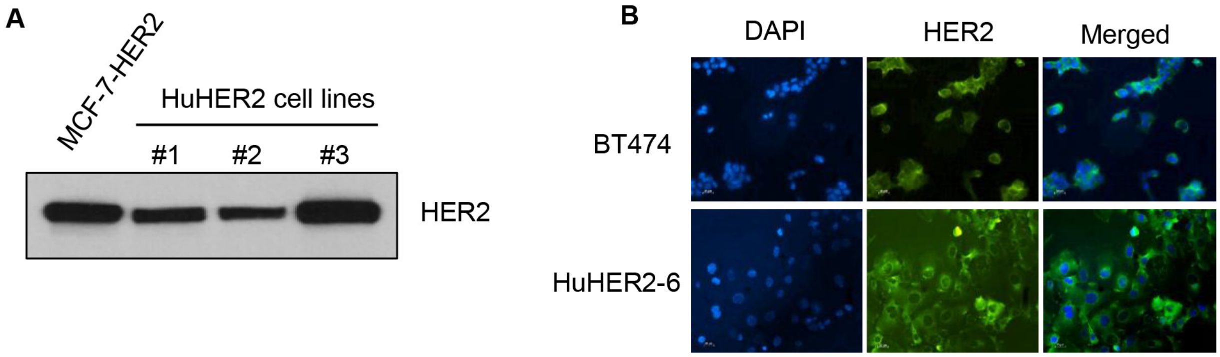

Supplementary Figure 2: Establishment of HuHER2 cell lines stably overexpressing Human HER2. **(A)** Western blot analysis of HER2 using anti-HER2 antibody in three HuHER2 cell lines. **(B)** Immunofluorescence analysis of HER2 in HuHER2 cell lines by FITC conjugated transtuzumab.

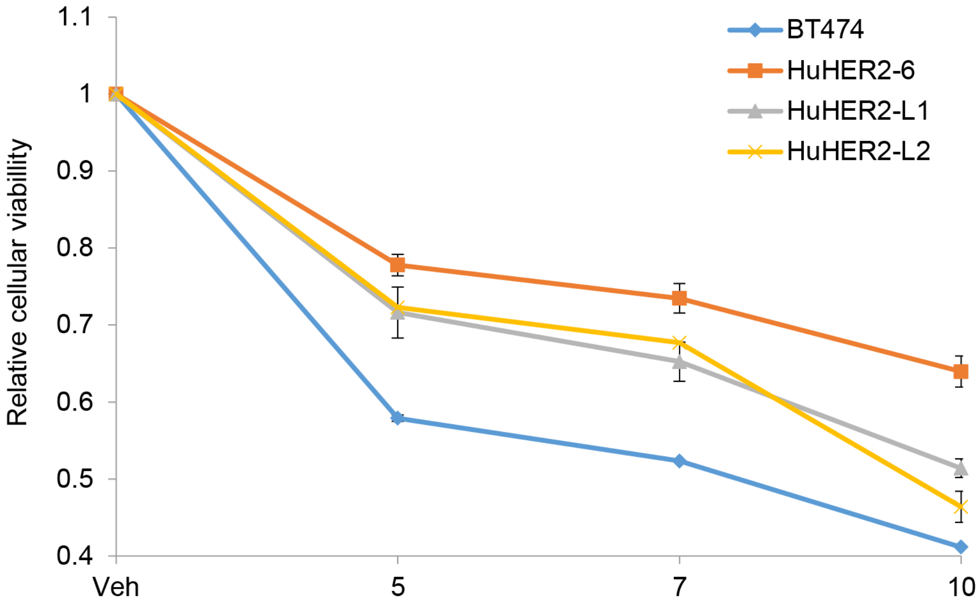

Supplementary Figure 3: Cellular viability assay of HuHER2 cell lines exposed to trastuzumab. MTT assay was performed in BT474, HuHER2-6, HuHER2-L1 and L2 cells treated with 5, 7 and 10 μg/ml of trastuzumab for 3 days.

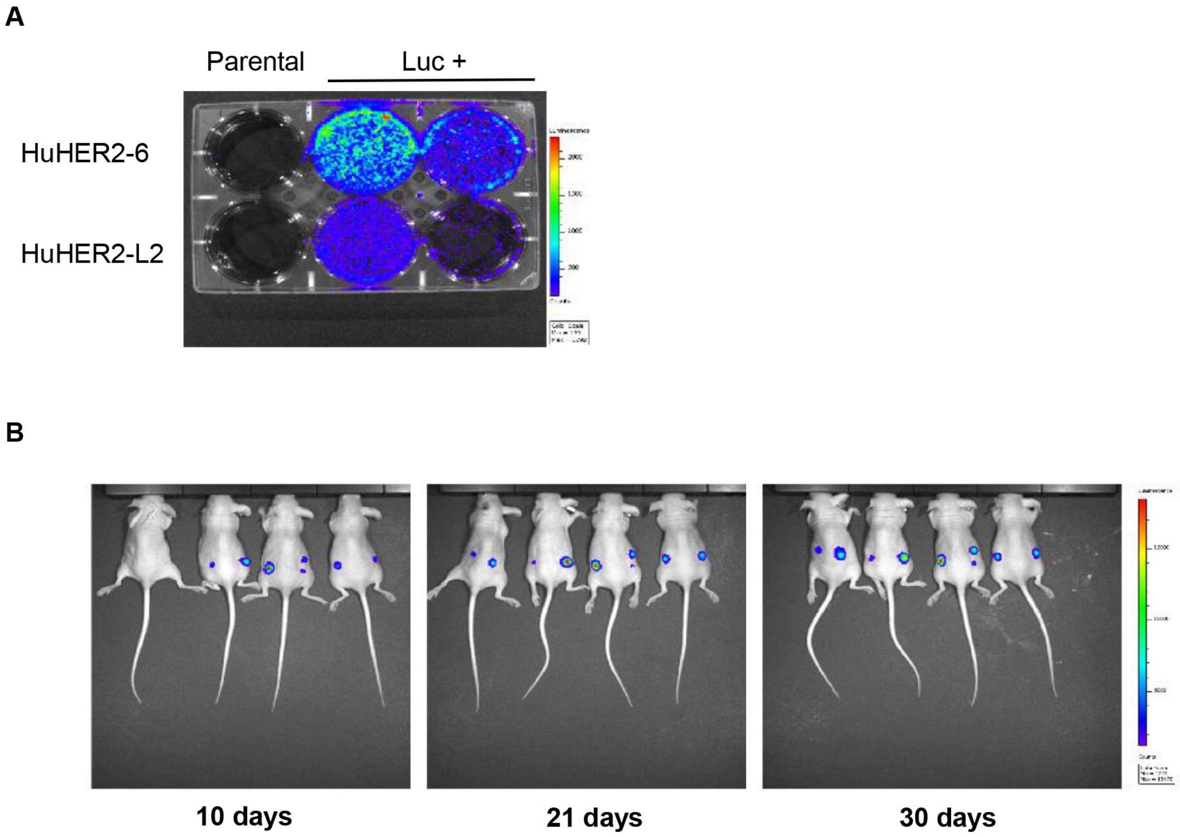

Supplementary Figure 4: Establishment of luciferase reporter stable HuHER2-L2-Luc+ cell line. **(A)** Bioluminescence imaging of HuHER2 cell lines in a plate. **(B)** Bioluminescence imaging of athymic nude mice orthotopically implanted with HuHER2-L2-Luc+ cells.

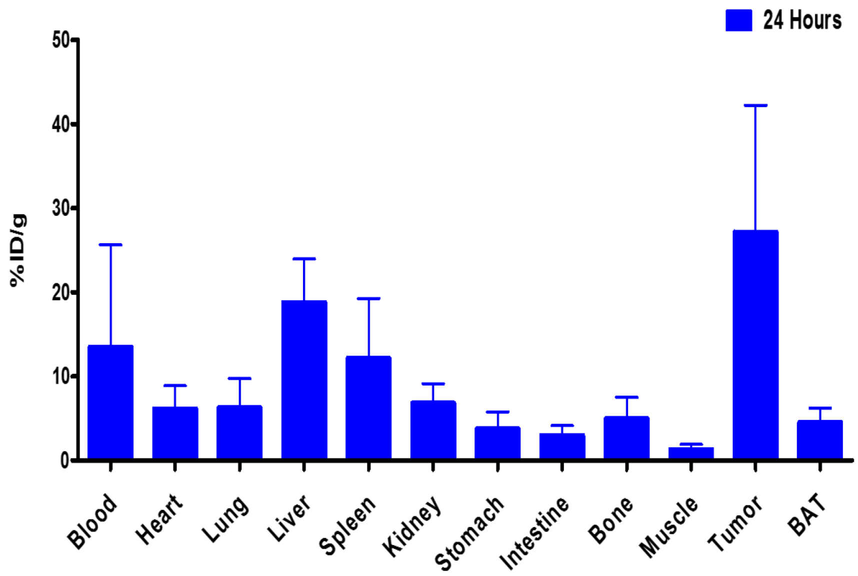

Supplementary Figure 5: Biodistribution of ^{111}In-DTPA-trastuzumab in HuHER2-L2-Luc+ cell tumor-bearing athymic nude mice at 24 hours p.i.

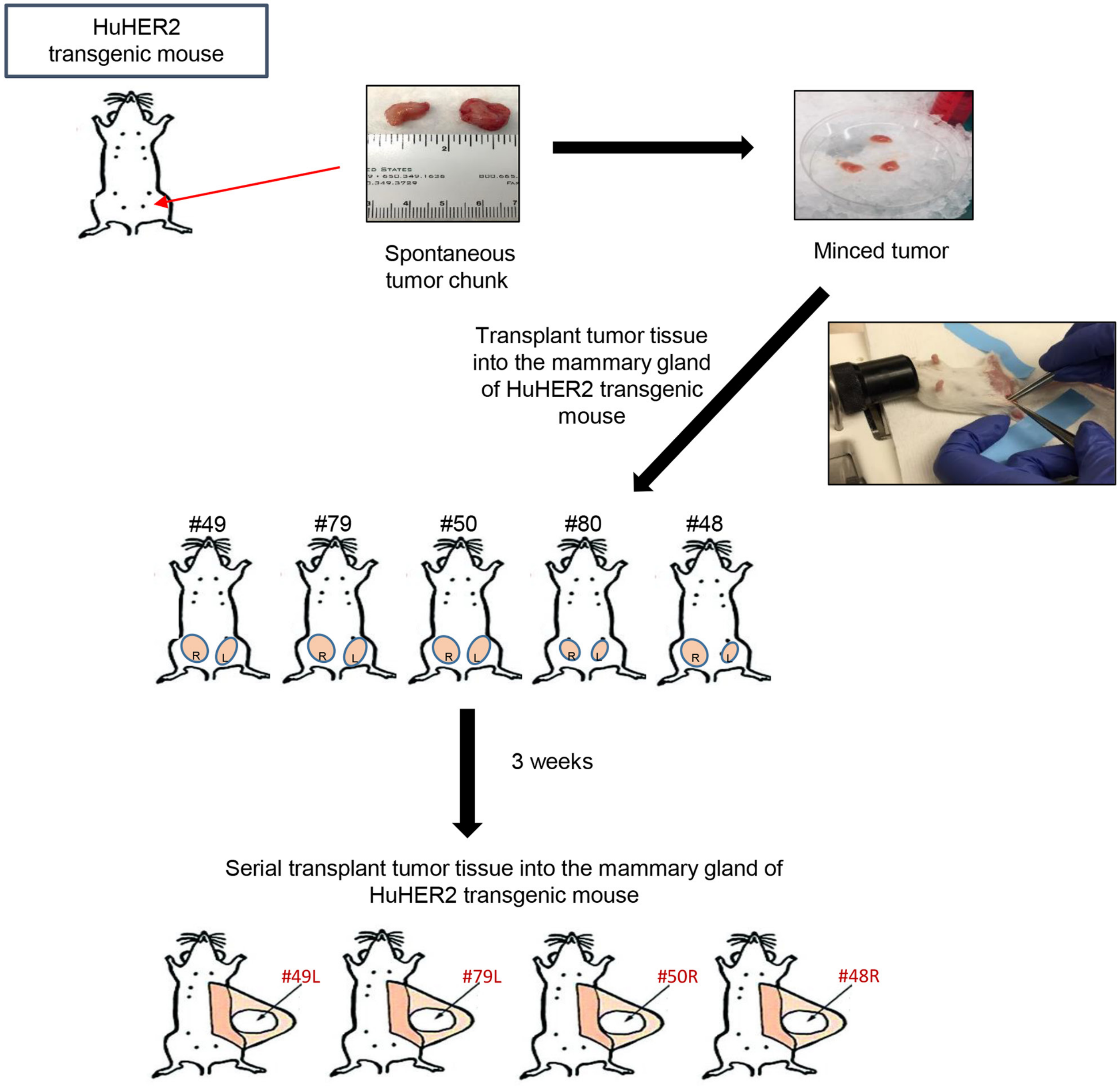

Supplementary Figure 6: Schematic diagram of the experimental procedure in serial transplantation of HuHER2 tumor tissues.
